# Patient-Centered Economic Burden of Diabetic Macular Edema: Retrospective Cohort Study

**DOI:** 10.2196/56741

**Published:** 2024-10-08

**Authors:** Kyungseon Choi, Sang Jun Park, Hyuna Yoon, Seoyoon Choi, Yongseok Mun, Seok Kim, Sooyoung Yoo, Se Joon Woo, Kyu Hyung Park, Junghyun Na, Hae Sun Suh

**Affiliations:** 1 Department of Regulatory Science Graduate School Kyung Hee University Seoul Republic of Korea; 2 Institute of Regulatory Innovation through Science Kyung Hee University Seoul Republic of Korea; 3 Department of Ophthalmology Seoul National University College of Medicine Seoul National University Bundang Hospital Seongnam Republic of Korea; 4 Department of Ophthalmology Kangnam Sacred Heart Hospital Hallym University College of Medicine Seoul Republic of Korea; 5 Healthcare ICT Research Center Office of eHealth Research and Businesses Seoul National University Bundang Hospital Seongnam Republic of Korea; 6 Department of Ophthalmology Seoul National University College of Medicine Seoul National University Hospital Seoul Republic of Korea; 7 College of Pharmacy Kyung Hee University Seoul Republic of Korea

**Keywords:** diabetic macular edema, economic burden, cost of illness, retrospective cohort study, patient-centered care, Observational Medical Outcomes Partnership Common Data Model

## Abstract

**Background:**

Diabetic macular edema (DME), a leading cause of blindness, requires treatment with costly drugs, such as anti–vascular endothelial growth factor (VEGF) agents. The prolonged use of these effective but expensive drugs results in an incremental economic burden for patients with DME compared with those with diabetes mellitus (DM) without DME. However, there are no studies on the long-term patient-centered economic burden of DME after reimbursement for anti-VEGFs.

**Objective:**

This retrospective cohort study aims to estimate the 3-year patient-centered economic burden of DME compared with DM without DME, using the Common Data Model.

**Methods:**

We used medical data from 1,903,603 patients (2003-2020), transformed and validated using the Observational Medical Outcomes Partnership Common Data Model from Seoul National University Bundang Hospital. We defined the group with DME as patients aged >18 years with nonproliferative diabetic retinopathy and intravitreal anti-VEGF or steroid prescriptions. As control, we defined the group with DM without DME as patients aged >18 years with DM or diabetic retinopathy without intravitreal anti-VEGF or steroid prescriptions. Propensity score matching, performed using a regularized logistic regression with a Laplace prior, addressed selection bias. We estimated direct medical costs over 3 years categorized into total costs, reimbursement costs, nonreimbursement costs, out-of-pocket costs, and costs covered by insurance, as well as healthcare resource utilization. An exponential conditional model and a count model estimated unbiased incremental patient-centered economic burden using generalized linear models and a zero-inflation model.

**Results:**

In a cohort of 454 patients with DME matched with 1640 patients with DM, the economic burden of DME was significantly higher than that of DM, with total costs over 3 years being 2.09 (95% CI 1.78-2.47) times higher. Reimbursement costs were 1.89 (95% CI 1.57-2.28) times higher in the group with DME than with the group with DM, while nonreimbursement costs were 2.54 (95% CI 2.12-3.06) times higher. Out-of-pocket costs and costs covered by insurance were also higher by a factor of 2.11 (95% CI 1.58-2.59) and a factor of 2.01 (95% CI 1.85-2.42), respectively. Patients with DME had a significantly higher number of outpatient (1.87-fold) and inpatient (1.99-fold) visits compared with those with DM (*P*<.001 in all cases).

**Conclusions:**

Patients with DME experience a heightened economic burden compared with diabetic patients without DME. The substantial and enduring economic impact observed in real-world settings underscores the need to alleviate patients’ burden through preventive measures, effective management, appropriate reimbursement policies, and the development of innovative treatments. Strategies to mitigate the economic impact of DME should include proactive approaches such as expanding anti-VEGF reimbursement criteria, approving and reimbursing cost-effective drugs such as bevacizumab, advocating for proactive eye examinations, and embracing early diagnosis by ophthalmologists facilitated by cutting-edge methodologies such as artificial intelligence for patients with DM.

## Introduction

### Background

Diabetic retinopathy (DR) is a common complication of diabetes mellitus (DM) that affects the eyes and can lead to vision loss or blindness [1-3]. Nonproliferative DR (NPDR) is an early stage of DR that causes small leaks in blood vessels and changes in the retina. As NPDR progresses to proliferative DR (PDR), new abnormal blood vessels grow, causing severe vision loss or blindness. Patients with DR can develop diabetic macular edema (DME) at any stage, even in the early stage of NPDR. DME is characterized by macular thickening resulting from fluid accumulation in the macula due to DR. DME is the most common cause of vision loss in patients with DR [4-6]. As DME management is crucial to preventing severe vision loss, active treatment options for DME include the use of intravitreal anti–vascular endothelial growth factor (VEGF) agents, intraocular steroids, focal laser photocoagulation, and vitrectomy. On the basis of a network meta-analysis of clinical trials, anti-VEGF agents are recommended as the primary treatment for DME [7], but they can be costly. While certain anti-VEGF drugs, including aflibercept, ranibizumab, faricimab, and brolucizumab, are eligible for reimbursement, the high cost of these medications, coupled with copayment rates at tertiary hospitals and stringent reimbursement criteria, imposes a substantial economic burden on patients. Another anti-VEGF drug, bevacizumab, is also recommended in clinical guidelines [8,9] and is cost-effective for treating DME [10]. However, it is neither approved nor reimbursed in the United Kingdom, South Korea, and other countries.

Patients with PDR are treated with anti-VEGF agents regardless of the presence of DME and have a high disease burden. No treatment is necessary for NPDR without DME, and patients with NPDR without DME may not have a significant economic burden. However, in NPDR with DME, treatment is typically prioritized for DME, and compared with patients with DM, patients with NPDR and DME may face a considerable economic burden owing to the high cost of treatment and frequent visits to tertiary hospitals, resulting in high copayment rates and costly nonreimbursable items.

Understanding the direct medical healthcare costs and healthcare resource utilization (HRU) associated with managing these conditions, including nonreimbursement and out-of-pocket costs, is crucial for developing cost-effective strategies for DR prevention and management. Specifically, estimating the economic burden and HRU forms a crucial basis for allocating public health resources and making informed decisions on new drug reimbursement, including economic evaluations and budget impact analyses in health policy. Numerous studies on the economic burden of disease have been published in various formats, such as policy reports and academic papers [11-14], providing essential evidence for policy makers. However, although there are studies detailing the direct treatment costs of patients with DME (eg, the study by Gascon at al [15], which found that the mean total treatment cost per eye in France was US $5048 over 1 year, with a range of US $1260-$9230 [with a currency exchange rate of €1=US $1.12]), there remains a significant gap in well-designed research examining the long-term economic burden of DME from the patients’ perspective.

### Objectives

This retrospective cohort study aimed to estimate the economic burden of patients with DME compared with that of patients with DM without DME, using the Observational Health Data Sciences and Informatics (OHDSI) Observational Medical Outcomes Partnership (OMOP) Common Data Model (CDM). Real-world data on DME are challenging to define using claims data because diagnosis codes do not exist, and treatment overlaps with that of PDR or other ophthalmic diseases. By analyzing standardized data from the electronic medical records (EMRs) of patients diagnosed with DME, we estimated the direct medical healthcare costs associated with managing DME, particularly from a patient-centered perspective, including nonreimbursement and out-of-pocket costs.

## Methods

### Study Design and Data Source

We conducted a retrospective cohort study using EMR data from Seoul National University Bundang Hospital (SNUBH), standardized to the OMOP CDM by the SNUBH Healthcare ICT Research Center. SNUBH is the first stage 7 hospital outside of North America and the first fully digital hospital with an EMR system in South Korea. The EMR data, including laboratory results, nonreimbursement items, and cost data, were extracted, transformed, and loaded into the OMOP CDM (version 5.3), following OHDSI guidelines [16]. The data set included 1,903,603 patients, with data spanning from April 2003 to December 2020. All data were verified using Automated Characterization of Health Information at Large-Scale Longitudinal Evidence Systems [17] and double-checked by data analysts and clinicians.

This study adheres to the Strengthening the Reporting of Observational Studies in Epidemiology (STROBE) guidelines.

### Study Population

**Figure 1:**
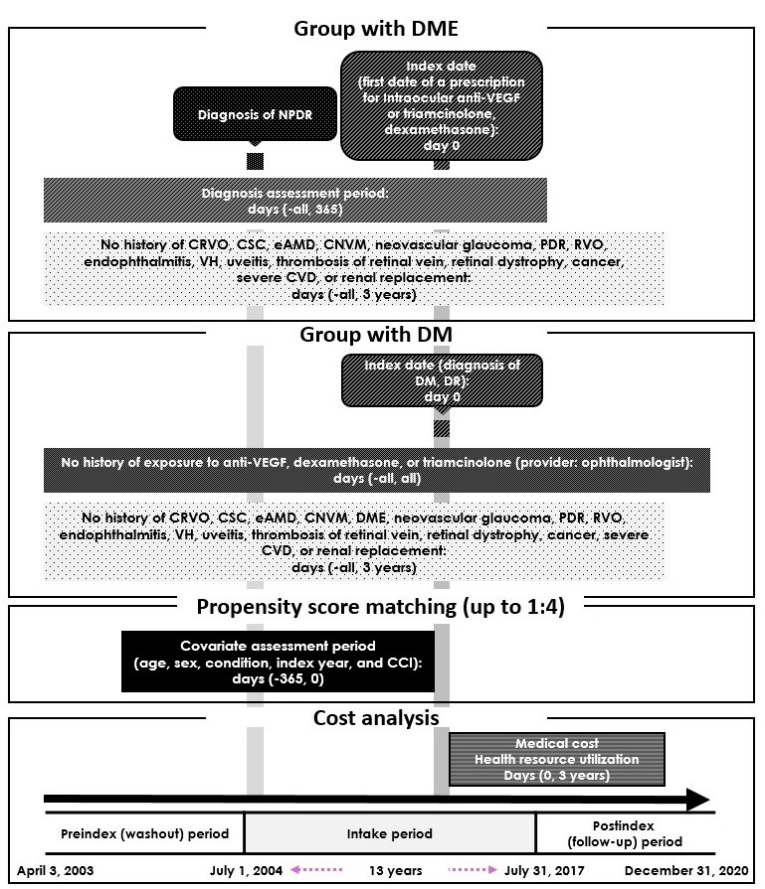
Analysis scheme. Data from April 2003 to December 2020 were included. The intake period was defined as July 1, 2004, to July 31, 2017. The follow-up period was 3 years from the index date. CCI: Charlson Comorbidity Index; CNV: choroidal neovascularization; CRVO: central retinal vein occlusion; CSC: central serous chorioretinopathy; CVD: cardiovascular disease; DM: diabetes mellitus; DME: diabetic macular edema; DR: diabetic retinopathy; eAMD: exudative age-related macular degeneration; NPDR: nonproliferative diabetic retinopathy; PDR: proliferative diabetic retinopathy; RVO: retinal vein occlusion; VEGF: vascular endothelial growth factor; VH: variceal hemorrhage.

We defined the eligibility criteria for patients with DME (group with DME) and those with DM without DME (group with DM). For a 3-year follow-up period and a 1-year washout period, we set the intake period from July 1, 2004, to July 31, 2017. We defined the index date for the group with DME as the first date of a prescription from an ophthalmologist for intraocular anti-VEGF, triamcinolone, or dexamethasone during the intake period. To accurately identify patients with DME, we defined patients with DME as those who had at least 1 occurrence of NPDR before the index date or 1 year after the index date; we excluded patients with other ophthalmic diseases requiring intraocular anti-VEGF or steroid treatment (ie, choroidal neovascularization, central serous chorioretinopathy, exudative age-related macular degeneration, PDR, retinal vein occlusion, variceal hemorrhage, neovascular glaucoma, endophthalmitis, uveitis, thrombosis of the retinal vein, and retinal dystrophy). For the group with DM, the index date was defined as the date of diagnosis of DM or DR. Patients with DM who had a prescription for intraocular anti-VEGF, triamcinolone, or dexamethasone, as well as those with an occurrence of NPDR or other ophthalmic diseases requiring intraocular anti-VEGF or steroid treatment, were excluded. To prevent overfitting of the model by outliers, we excluded patients with cancer, renal replacement, or severe cardiovascular disease (ie, cerebrovascular accident, ischemic heart disease, and acute heart disease) in both groups (Figure 1).

### Outcomes

To assess the economic burden of patients with DME, our primary outcomes focused on direct medical costs incurred at the hospital over 3 years after the index date. We categorized the direct medical and incremental costs of the group with DME into 5 segments: total costs, reimbursement costs, nonreimbursement costs, out-of-pocket costs, and costs covered by insurance (Textbox 1). Total medical costs were calculated as the sum of reimbursement and nonreimbursement costs or the sum of costs covered by insurance and out-of-pocket costs. We further analyzed total medical costs based on reimbursement categorization and payment entities. For reimbursement categorization, we calculated reimbursement and nonreimbursement costs for each relevant item, irrespective of the payer. To estimate medical costs by payment entities, costs covered by insurance included all costs from national health insurance services, insurance contractors, and employers. Out-of-pocket costs encompassed all expenses directly incurred by the patients. All costs were measured in Korean won and subsequently converted to US dollars. A currency exchange rate of 1200 Korean won=US $1 is applicable.

**Definitions of cost categories for the patient-centered economic burden outcome.**

- Total medical cost: all expenses associated with each patient in the hospital, encompassing both reimbursed and nonreimbursed expenditures over 3 years (the sum of the reimbursement costs and nonreimbursement costs or the sum of costs covered by insurance and out-of-pocket costs)
- Reimbursement cost: all expenses incurred by patients restricted to items eligible for reimbursement over 3 years
- Nonreimbursement cost: all expenses incurred by patients restricted to items not eligible for reimbursement over 3 years
- Costs covered by insurance: all expenses paid by national health insurance services, insurance contractors, and employers over 3 years
- Out-of-pocket cost: all expenses borne directly by the patient, irrespective of any reimbursement, over 3 years

As secondary outcomes, we analyzed medical costs by follow-up date and HRU. The medical costs by follow-up date were calculated as the average costs per group by day after index date and the accumulated costs by follow-up date. HRU was estimated in 3 categories over 3 years, regardless of specialty: count of outpatient visits and inpatient visits as well as length of stay (LOS) for inpatients.

To estimate the medical costs by follow-up date, we used the following formula (equation 1):

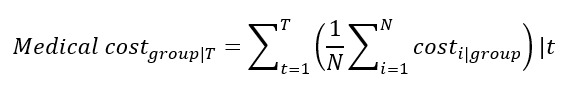



where *Medical cost* is the accumulated cost by *group* at day *T*, *cost* denotes the medical cost at day *t* by *group*, *i* indexes the individual in the treatment group (either DME or DM), and *t* indexes the time in day.

To assess bias after propensity score matching, negative control outcomes were used. A total of 598 covariates, such as vedolizumab, zolpidem, zinc bromide, zafirlukast, wrist drop, and tramadol, were included as negative control outcomes.

### Statistical Analysis

Data analyses were conducted using ATLAS (version 2.10.1; OHDSI); a health resources econometric analysis tool (HERMES; version 0.1.0; OHDSI); and HADES (previously the OHDSI Methods Library; OHDSI), a collection of open-source R packages that offer functions that can be used together to perform a complete observational study, starting from data in the CDM, and resulting in estimates and supporting statistics, figures, and tables. To compare the 2 groups, we evaluated the baseline characteristics of the patients and performed propensity score matching with a ratio of up to 1:4, using regularized logistic regression with a Laplace prior (which is equivalent to least absolute shrinkage and selection operator [LASSO]). Baseline patient characteristics such as biological sex, age group, index year, condition, and Charlson Comorbidity Index (CCI) were used as covariates for the propensity score matching model, while highly correlated covariates such as anti-VEGF and NPDR were excluded. After propensity score matching, we considered covariates with a standardized mean difference of ≥0.10 between the 2 groups as unmatched covariates and included them in the exponential conditional model (ECM). If the hazard ratio for negative control outcomes included 1 at the 75% CI, we considered that there was no selection bias. To prevent distortion and overfitting of the model, we defined groups without outliers, excluding zero-cost patients (those who did not incur any cost due to their participation in clinical trials) in groups. For the primary outcome, HERMES was used for the ECM to estimate the precise cost, with adjustments for confounders and positive skewness [18]. In the ECM, the gamma distribution and log link function were determined by modified Park tests, Box-Cox tests, and goodness-of-fit evaluations using the Akaike information criterion and Bayesian information criterion [19]. We performed descriptive analyses as a sensitivity analysis. Among the secondary outcomes, count outcomes such as HRU over 3 years were analyzed using HERMES with Poisson or negative binomial models. Age, sex, preindex cost, and unmatched covariates from propensity score matching were considered covariates in the ECM and count model, including the group variable. If the observed median was 0, a zero-inflation model was considered, and the covariates were selected by backward elimination for addressing the convergence issue. We assumed that there were no missing data for covariates before matching and that any missing data for outcomes and follow-up loss due to other diseases were similar between the groups as a result of propensity matching. The medical costs by follow-up date were suggested as a cumulative average daily increase for each group after propensity score matching and outlier removal.

### Ethical Considerations

This study was approved by the institutional review board of SNUBH (X-2012-657-902). The data were deidentified and anonymized; therefore, informed consent was not required. As our study involved secondary data analysis, there was no direct interaction with participants, and no compensation was provided.

## Results

### Patient Characteristics

**Figure 2:**
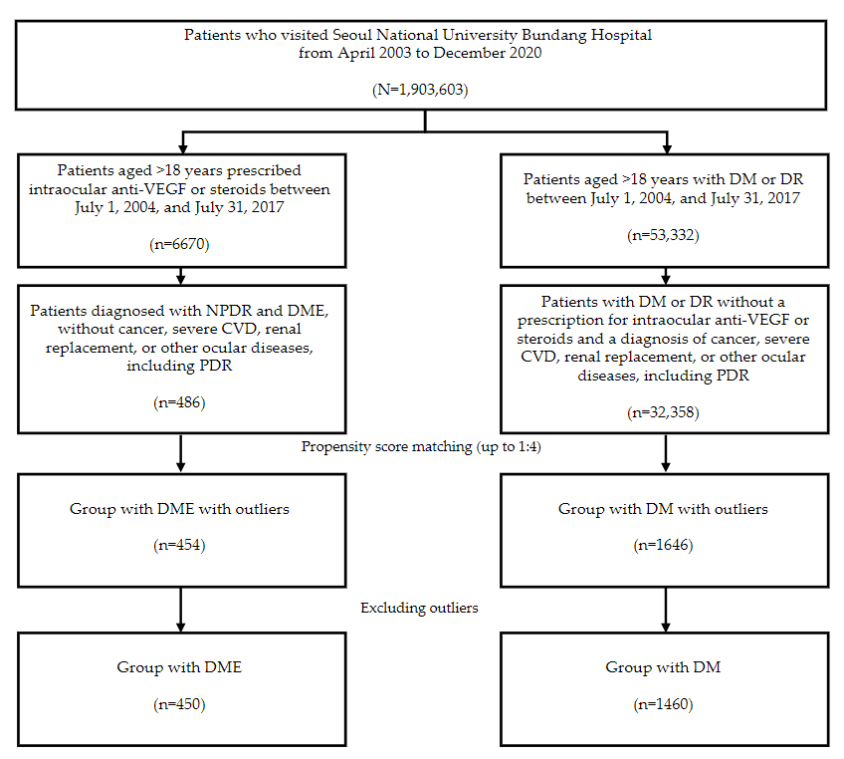
Selection flow of patients with diabetic macular edema (DME) and those with diabetes mellitus (DM) from April 2003 to December 2020. CVD: cardiovascular disease; DR: diabetic retinopathy; NPDR: nonproliferative diabetic retinopathy; PDR: proliferative diabetic retinopathy; VEGF: vascular endothelial growth factor.

Of the 1,903,603 patients who visited SNUBH between 2003 and 2020, we identified 32,844 (1.73%) who met the eligibility criteria (patients with NPDR and DME: n=486, 1.48%; patients with DM: n=32,358, 98.52%). After propensity score matching, we matched 454 (93.4%) of the 486 patients with DME with 1646 (5.06%) of the 32,358 patients with DM with outliers. Of the 454 patients in the group with DME, and the 1646 patients in the group with DM, 4 (0.9%) and 186 (11.3%) outliers were excluded respectively, leaving 450 (99.1%) and 1460 (88.7%) patients (Figure 2).

In the propensity score model, we matched 140 covariates, and most of them (n=129, 92.1%) had a standardized mean difference of <0.10 between the group with DME and the group with DM (Table 1). However, 11 (7.9%) of the 140 variables, including the CCI, had a standardized mean difference of ≥0.10, indicating unmatched covariates, and were included in the ECM. Of the 598 negative control outcomes, events could be identified for only 4 (0.7%): impingement syndrome of the shoulder region, falls, hyperosmolality, and difficulty sleeping, with a hazard ratio of 1 within the 75% CI.

**Table 1:** Baseline demographic and clinical characteristics of the group with diabetic macular edema (DME) and the group with diabetes mellitus (DM) in the index period from July 2004 to July 2017 before and after propensity score (PS) matching (n=32,844).

Characteristics^a^		Before PS matching				After PS matching		
		Group with DME (n=486)	Group with DM (n=32,358)	SMD^b^	Group with DME (n=454)		Group with DM (n=1646)	SMD
Age (y), mean (SD)		64.97 (11.15)	58.21 (13.79)	0.54	64.57 (11.27)		64.99 (11.99)	–0.01
**Sex, n (%)**								
	Female	242 (49.79)	16,191 (50.04)	0.00	224 (49.34)		819 (51.12)	–0.04
Charlson Comorbidity Index: Romano adaptation, mean (SD)^c^		2.39 (1.18)	1.48 (0.88)	0.87	2.30 (1.12)		2.45 (1.27)	–0.19
**Medical history: general, n (%)^d^**								
	Acute respiratory disease	1 (0.21)	145 (0.45)	–0.04	1 (0.22)		2 (0.28)	–0.01
	Chronic liver disease	3 (0.62)	563 (1.74)	–0.10	3 (0.66)		14 (0.81)	–0.02
	Chronic obstructive lung disease	1 (0.21)	118 (0.36)	–0.03	1 (0.22)		9 (0.66)	–0.07
	Dementia	6 (1.23)	469 (1.45)	–0.02	5 (1.1)		19 (1.23)	–0.01
	Depressive disorder	6 (1.23)	323 (1)	0.02	6 (1.32)		22 (1.38)	0.00
	Gastroesophageal reflux disease	5 (1.03)	543 (1.68)	–0.06	5 (1.1)		20 (1.17)	–0.01
	Gastrointestinal hemorrhage	1 (0.21)	267 (0.83)	–0.09	1 (0.22)		8 (0.46)	–0.04
	Hyperlipidemia	18 (3.70)	2982 (9.22)	–0.23	17 (3.74)		43 (3.1)	0.04
	Hypertensive disorder	78 (16.05)	7998 (24.72)	–0.22	71 (15.64)		234 (14.87)	0.02
	Lesion of liver	2 (0.41)	579 (1.79)	–0.13	2 (0.44)		9 (0.53)	–0.01
	Osteoarthritis	13 (2.67)	1478 (4.57)	–0.10	12 (2.64)		42 (2.37)	0.02
	Pneumonia	3 (0.62)	495 (1.53)	–0.09	3 (0.66)		12 (0.83)	–0.02
	Renal impairment	16 (3.29)	663 (2.05)	0.08	16 (3.52)		80 (4.88)	–0.07
	Rheumatoid arthritis	4 (0.82)	304 (0.94)	–0.01	4 (0.88)		8 (0.44)	0.05
	Viral hepatitis C	3 (0.62)	91 (0.28)	0.05	1 (0.22)		1 (0.06)	0.04
**Medical history: cardiovascular disease, n (%)^d^**								
	Atrial fibrillation	5 (1.03)	536 (1.66)	–0.05	5 (1.1)		12 (0.66)	0.05
	Cerebrovascular disease	17 (3.5)	2389 (7.38)	–0.17	16 (3.52)		60 (3.69)	–0.01
	Coronary arteriosclerosis	8 (1.65)	761 (2.35)	–0.05	8 (1.76)		19 (1.05)	0.06
	Heart disease	15 (3.09)	2347 (7.25)	–0.19	14 (3.08)		49 (2.86)	0.01
	Heart failure	1 (0.21)	310 (0.96)	–0.10	1 (0.22)		10 (0.55)	–0.05
	Peripheral vascular disease	5 (1.03)	111 (0.34)	0.08	2 (0.44)		15 (0.84)	–0.05
**Unmatched covariates, n (%)^c,d^**								
	Rhegmatogenous retinal detachment	5 (1.03)	4 (0.01)	0.14	5 (1.1)		1 (0.06)	0.14
	Degeneration of macular and posterior pole	5 (1.03)	7 (0.02)	0.14	5 (1.1)		3 (0.17)	0.12
	Degeneration of posterior pole of eye	5 (1.03)	7 (0.02)	0.14	5 (1.1)		3 (0.17)	0.12
	Discharge from eye	5 (1.03)	10 (0.03)	0.14	5 (1.1)		3 (0.17)	0.12
	Obstruction of nasolacrimal duct	5 (1.03)	20 (0.06)	0.13	5 (1.1)		1 (0.06)	0.14
	Lesion of eyelid	4 (0.82)	17 (0.05)	0.12	4 (0.88)		1 (0.06)	0.12
	Hypertensive retinopathy	4 (0.82)	20 (0.06)	0.11	3 (0.66)		1 (0.06)	0.10
	Secondary glaucoma	3 (0.62)	7 (0.02)	0.11	3 (0.66)		1 (0.06)	0.10
	Labyrinthine disorder	3 (0.62)	297 (0.92)	–0.03	3 (0.66)		1 (0.06)	0.10
	Diabetic foot	3 (0.62)	144 (0.45)	0.02	3 (0.66)		34 (1.87)	–0.11

^a^These covariates are a subset of the 140 covariates used for PS matching.

^b^SMD: standardized mean difference.

^c^Unmatched covariates based on SMD, which were adjusted in the subsequent exponential conditional model and count model.

^d^Medical history and medication use were identified by diagnosis and prescription within 1 year before the index date.

### The Incremental Patient-Centered Economic Burden of DME Versus DM After Propensity Score Matching

The primary outcomes showed that the group with DME had significantly higher costs than the group with DM in all categories, including total costs, reimbursement costs, nonreimbursement costs, out-of-pocket costs, and costs covered by insurance after adjusting for remaining confounders with ECM (*P*<.001; Table 2). The total cost for the group with DME was US $2359 more than that for the group with DM (Table 3), which is 2.09 (95% CI 1.78-2.47) times higher. Reimbursement costs were 1.89 (95% CI 1.57-2.28) times higher in the DME group compared with the DM group (US $2854.25, standard error (SE) US $192.85 vs US $1525.78, SE US $100.76), while nonreimbursement costs were 2.54 (95% CI 2.12-3.06) times higher (US $1618.89, SE US $76.39 vs US $627.52, SE US $29.91). The out-of-pocket costs and costs covered by insurance were also higher in the DME group than the DM group by a factor of 2.11 (95% CI 1.58-2.59; US $1871.17, SE US $177.34 vs US $970.01, SE US $183.45) and a factor of 2.01 (95% CI 1.85-2.42; US $2593.32, SE US $114.72 vs US $1208.88, SE US $46.54), respectively. Age and preindex cost were positively correlated with all costs (^P^=.008 for nonreimbursement cost, ^P^<.001 for other costs), while the CCI was not significantly correlated with nonreimbursement costs but positively correlated with other costs (^P^=.005 for total costs, ^P^=.002 for reimbursement cost, ^P^=.03 for costs covered by insurance, and ^P^=.003 for out-of-pocket costs). not significantly correlated with nonreimbursement costs but positively correlated with other costs. All unmatched covariates were insignificant.

**Table 2:** The exponential conditional models over 3 years for the group with diabetic macular edema (DME) compared with the group with diabetes mellitus (DM) after propensity score matching in patients for estimating economic burden.

Exponential conditional model (coefficient)^a^	Total costs		Reimbursement costs		Nonreimbursement costs		Costs covered by insurance			Out-of-pocket costs	
	β	*P* value	β	*P* value	β	*P* value	β	*P* value	β		*P* value
Intercept	6.3596	<.001	5.7563	<.001	5.6721	<.001	4.8656	<.001	6.2848		<.001
Group^b^	0.7394	<.001	0.6364	<.001	0.9329	<.001	0.7005	<.001	0.7483		<.001
Age (y)^c^	0.0142	<.001	0.0169	<.001	0.0090	.008	0.0227	<.001	0.0078		.002
Sex^d^	0.1125	.11	0.1169	.15	0.1034	.19	0.1161	.28	0.1073		.07
Preindex cost (US $)^e^	0.00008	<.001	0.00009	<.001	0.00004	.006	0.0001	<.001	0.00005		<.001
Charlson Comorbidity Index	0.0840	.005	0.1071	.002	0.0285	.39	0.0968	.03	0.0737		.003
Rhegmatogenous retinal detachment	0.2098	.74	0.5303	.45	–0.5766	.41	0.7083	.45	–0.1739		.74
Degeneration of macula and posterior pole	–0.7872	.17	–1.1197	.09	–0.3211	.62	–1.5568	.07	–0.4344		.36
Discharge from eye	–0.5023	.35	–0.8284	.17	–0.0386	.95	–1.0515	.19	–0.1788		.69
Obstruction of nasolacrimal duct	–0.4781	.48	–0.5381	.49	–0.3361	.66	–0.1756	.89	–0.5049		.37
Lesion of eyelid	0.1415	.85	–0.0047	.99	0.3948	.63	–0.9050	.41	0.5853		.33
Hypertensive retinopathy	0.5335	.48	0.1840	.83	0.8669	.31	–0.0904	.94	0.7296		.24
Secondary glaucoma	0.0065	.99	–0.0738	.93	0.1137	.90	–0.3135	.79	0.1010		.87
Labyrinthine disorder	0.1522	.84	0.0551	.95	0.3323	.70	0.0740	.95	0.2516		.69
Diabetic foot	0.2552	.37	0.1356	.67	0.5071	.11	0.2031	.63	0.3030		.20

^a^The exponential conditional models were conducted using a log link function with gamma distribution excluding outliers.

^b^The reference group was the group with DM.

^c^Age was included as a continuous variable.

^d^Sex was represented as a binary variable, with 1 indicating *male* and 0 indicating *female*.

^e^Preindex cost was calculated for 1 year before the index date.

**Table 3:** The estimated incremental healthcare costs over 3 years for the group with diabetic macular edema (DME) compared with the group with diabetes mellitus (DM) after the index date from the exponential conditional models after propensity score matching in patients. A currency exchange rate of 1200 Korean won=US $1 is applicable.

Exponential conditional model (costs; US $)^a^, mean (SE)	Total costs	Reimbursement costs	Nonreimbursement costs	Costs covered by insurance	Out-of-pocket costs
Group with DME (n=450^b^)	4502.52 (251.79)	2854.25 (192.85)	1618.89 (76.39)	1871.17 (177.34)	2593.32 (114.72)
Group with DM (n=1460^b^)	2142.56 (109.13)	1525.78 (100.76)	627.52 (29.91)	970.01 (183.45)	1208.88 (46.54)
ΔCost	2359.96	1328.48	991.38	901.15	1384.44

^a^The exponential conditional models were conducted using a log link function with gamma distribution. The SEs of the estimated costs were calculated using bootstrapping.

^b^After propensity score matching, outliers were excluded.

### The Accumulative Cost and HRU for DME After Propensity Score Matching

**Figure 3:**
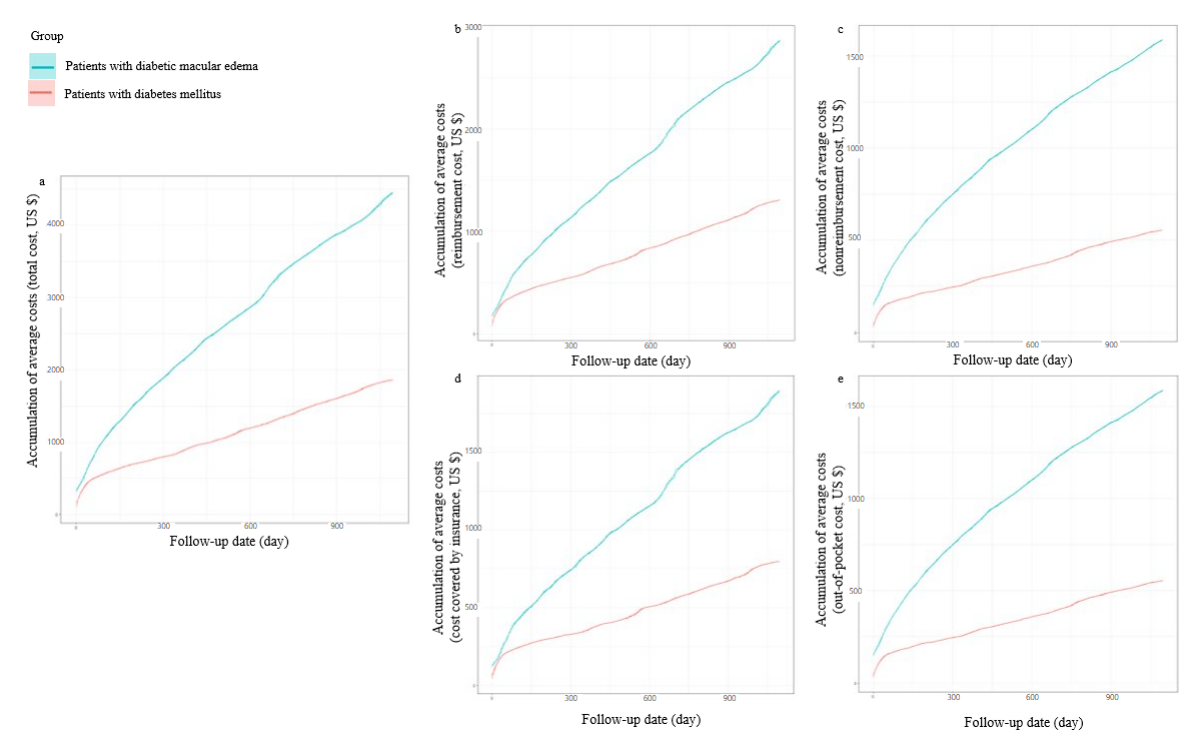
Accumulative costs by follow-up date over the 3-year follow-up period for patients with diabetic macular edema compared with those for patients with diabetes mellitus. (A) Total costs. (B) Reimbursement costs. (C) Nonreimbursement costs. (D) Costs covered by insurance. (E) Out-of-pocket costs.

For the secondary outcomes, the accumulative cost differences between the group with DME and the group with DM gradually increased over time for all cost categories. The difference between the groups became more substantial as the follow-up date progressed, particularly for nonreimbursement and out-of-pocket costs (Figure 3). Furthermore, the group with DME had higher average annual costs in years 1, 2, and 3 for all cost categories than the group with DM (Multimedia Appendix 1).

When comparing HRU over 3 years as secondary outcomes—the mean annual HRU for years 1, 2, and 3—we found that the group with DME had more outpatient and hospitalization visits than the group with DM (Multimedia Appendix 2); furthermore, the mean annual HRU provided by ophthalmologist for outpatient and inpatient visits for years 1, 2, and 3 was also higher in the group with DME than in the group with DM (Multimedia Appendix 3). Overdispersion was observed across all cost categories in the Poisson model (Pearson chi-square values ranging from 1.5 to >10). In the negative binomial model, the group with DME showed 1.87 (95% CI 1.66-2.12) times higher outpatient visits (Table 4). Outpatient visits for 3 years were 18.40 (SE 0.93) in the group with DME and 9.72 (SE 0.32) in the group with DM (Table 5). Age, CCI, and preindex cost were significantly positively associated with the number of outpatient visits (*P*=.02, .002, <.001, respectively). For inpatient visits and LOS, a zero-inflation negative binomial model was adapted, and the group with DME had 1.99 (95% CI 1.49-2.67) times higher inpatient visits over 3 years than the group with DM (*P*<.001). The number of inpatient visits was 0.44 (SE 0.04) in the group with DME and 0.25 (SE 0.02) in the group with DM. No between-group significance was observed for the LOS.

**Table 4:** The count models of the group with diabetic macular edema (DME) compared with that of the group with diabetes mellitus (DM) over 3 years after propensity score matching in patients for estimating healthcare resource utilization.

Count model (coefficient)^a^	Outpatient visits (n): count model		Inpatient visits (n)^b^						Length of stay (d)^b^				
			Zero-inflation model		Count model			Zero-inflation model				Count model	
	β	*P* value	β	*P* value	β	*P* value	β			*P* value	β		*P* value
Intercept	1.7220	<.001	0.3053	.66	–1.8849	<.001	1.3035			.02	–0.2211		.62
Group^c^	0.6257	<.001	0.8670	.001	0.6915	<.001	0.0933			.71	0.0767		.71
Age (y)^d^	0.0054	.02	0.0022	.82	0.0159	.004	–0.0068			.39	0.0188		.002
Sex^e^	–0.0301	.57	–0.1705	.52	0.1828	.21	–0.3052			.14	0.1148		.55
Preindex cost (US $)^f^	0.00004	<.001	–0.0008	<.001	0.00002	—^g^	–0.0008			<.001	0.00005		.05
Charlson Comorbidity Index	0.0706	.002	—	—	—	—	—			—	—		—
Rhegmatogenous retinal detachment	–0.6900	.15	—	—	—	—	—			—	—		—
Degeneration of macula and posterior pole	–0.6478	.14	—	—	—	—	—			—	—		—
Discharge from eye	0.1973	.62	—	—	—	—	—			—	—		—
Obstruction of nasolacrimal duct	–0.3816	.45	—	—	—	—	—			—	—		—
Lesion of eyelid	0.0777	.89	—	—	—	—	—			—	—		—
Hypertensive retinopathy	0.4509	.42	—	—	—	—	—			—	—		—
Secondary glaucoma	–0.2024	.73	—	—	—	—	—			—	—		—
Labyrinthine disorder	0.6377	.25	—	—	—	—	—			—	—		—
Diabetic foot	–0.0606	.78	—	—	—	—	—			—	—		—

^a^The count models were conducted using a log link function with negative binomial distribution excluding outliers.

^b^As the observed median was 0, a zero-inflation model was applied and estimated using a logit link function with binomial distribution. Covariates were selected by backward elimination due to the lack of variation among groups.

^c^The reference group was the group with DM.

^d^Age was included as a continuous variable.

^e^Sex was represented as a binary variable, with 1 indicating *male* and 0 indicating *female*.

^f^Preindex cost was calculated for 1 year before the index date. A currency exchange rate of 1200 Korean won=US $1 is applicable.

^g^Not applicable (variables were removed by backward elimination to address the convergence issue).

**Table 5:** The estimated incremental healthcare resource utilization of the group with diabetic macular edema (DME) compared with that of the group with diabetes mellitus (DM) over 3 years after the index date from the count models after propensity score matching in patients.

Count model^a^ (count), mean (SE)	Outpatient visits (n): count model	Inpatient visits (n)^b^		Length of stay (d)^b^	
		Zero-inflation model	Count model	Zero-inflation model	Count model
Group with DME (n=454^c^)	18.4038 (0.9328)	—^d^	0.4383 (0.0426)	—	1.8843 (0.3720)
Group with DM (n=1460^c^)	9.7246 (0.3190)	—	0.2480 (0.0158)	—	1.4148 (0.4037)
ΔCount	8.6792	—	0.1903	—	0.4695

^a^The count models were conducted using negative binomial distribution, and the SEs were calculated using bootstrapping.

^b^As the observed median was 0, a zero-inflation model was applied and estimated using a logit link function with binomial distribution. Covariates were selected by backward elimination due to the lack of variation among groups.

^c^After propensity score matching, outliers were excluded.

^d^Not applicable.

## Discussion

### Principal Findings

This retrospective cohort study evaluated the economic burden of DME using real-world data. The economic burden of DME was significantly higher than that of DM without DME (*P*<.001 in all cases). Comparing the total costs over 3 years using the ECM, we found that the total cost for the group with DME was 2.09 times higher. In particular, the economic burden on patients was greater than the coverage provided by insurance or reimbursement due to nonreimbursement items. Reimbursement costs and costs covered by insurance were 1.89 and 2.01 times higher, respectively, while nonreimbursement and out-of-pocket costs, representing the direct economic burden of patients, were 2.54 and 2.11 times higher, respectively. Age and preindex cost were significantly and positively associated with all costs, and CCI was significantly and positively associated with all costs, except nonreimbursement costs. When compared by follow-up period, patients with DME had consistently higher costs, not only in year 1 but also in years 2 and 3. The results of the count model show that the number of outpatient and inpatient visits over 3 years was also significantly higher in the group with DME than in the group with DM (*P*<.001 in all cases).

### Comparison With Previous Studies

As ophthalmic conditions significantly contribute to the medical costs associated with diabetes [14], and diabetic eye diseases can lead to vision loss or blindness with a high economic burden for patients, some previous studies have examined the burden of diabetic ophthalmic conditions such as DME and DR [15,20-28]. However, these studies were unable to estimate the patient-centered economic burden of DME due to several limitations: (1) difficulty in distinguishing the analyzed population, which included a mix of patients with DME and those with DR, including those with PDR without DME or NPDR without DME, as well as other ophthalmic conditions, due to the lack of a specific DME diagnosis in International Classification of Diseases, Tenth Revision, and the overlap in treatment modalities between PDR and DME [25,26]; (2) the use of outdated data before the introduction of anti-VEGF treatments and reliance on descriptive analysis rather than evaluating the marginal economic burden with a quasi-experimental control [20-23,29]; (3) short analysis periods of only 1 year [15,24]; and (4) a focus on reimbursed measurements as well as drugs such as aflibercept and ranibizumab, while excluding patient and physician choice of nonreimbursed drugs such as bevacizumab, leading to an underestimation of the patient burden [15,26]. Thus, while previous studies have highlighted the high descriptive economic burden of DR or DME, they have faced challenges in accurately estimating the patient-centered long-term economic burden, specifically for patients with DME. Our study has several strengths. First, we precisely defined patients with DME, excluding those with PDR, using EMR data converted to the OMOP CDM. We validated the clinical and cost data with clinicians, data scientists, and economists. This is the only real-world data set that met fit-for-use criteria, including nonreimbursed medications, measurements, and ophthalmology information. Using EMR data converted to the OMOP CDM, we not only defined patients with DME but also analyzed the economic burden from their perspective. Second, we implemented LASSO and ECM models to estimate the marginal patient burden, minimizing selection bias and confounders. Third, we compared multiple categories of long-term (3-year) direct medical costs between patients with DME and those with DM. With these strengths, our study provides valuable real-world evidence by demonstrating a significant incremental patient-centered economic burden for patients with DME, including nonreimbursed care, compared with those with DM.

### Policies to Address the Economic Burden of DME

Effective management strategies are crucial for patients with DME to prevent disease progression to PDR or vision loss, which is associated with a higher economic burden and a poorer prognosis [25,30]. The high costs of medications, coupled with unreimbursed and out-of-pocket expenses, may create economic burdens for patients with DME, potentially leading to hesitation in seeking treatment. To prevent a greater treatment burden in the future, active reimbursement policies, such as relaxing reimbursement criteria or reimbursing bevacizumab, along with comprehensive disease prevention and management strategies, should be considered. In the United States, bevacizumab is reimbursed for patients through the Centers for Medicare & Medicaid Services, ensuring broader access to this treatment option and reducing the financial burden on patients. However, in central and eastern Europe, as well as in other parts of the world, there is a lack of clinical practice guidelines for DME, and the accessibility and reimbursement of anti-VEGF treatments differ widely by region [31]. This discrepancy results in many patients remaining untreated or receiving inappropriate treatment. Similarly, in the United Kingdom and South Korea, bevacizumab is not reimbursed or even licensed. In South Korea, unlike other similarly treated conditions, such as exudative age-related macular degeneration, there is no special calculation system for exempted health insurance policies to reduce the burden on patients. These limitations highlight the urgent need for appropriate economic equity and the development of reimbursement policies to address the high out-of-pocket costs experienced by patients with DME. In addition to these policies, new drugs and technologies need to be developed to relieve the patient burden. The development and reimbursement of novel drugs have the potential to significantly improve patient outcomes, leading to better prognosis, enhanced quality of life, and reduced economic burden [32]. In addition, leveraging artificial intelligence for eye examinations conducted by ophthalmologists can facilitate the early detection and treatment of patients with DR, offering promising avenues for efficient and cost-effective care [28].

Furthermore, the current reimbursement policies regulating expensive drug costs might exacerbate patients’ clinical and economic burdens. A narrow drug reimbursement pathway, rather than a stringent drug authorization process, combined with an international reference pricing system, can lead to pharmaceutical companies skipping or withholding reimbursement in low-income countries. Consequently, patients in these countries may be compelled to pay for the drug out of pocket, thereby worsening their economic and clinical burdens. Addressing this issue requires various policy interventions, one of which involves implementing a 2-stage reimbursement process, with a revalidation process using real-world data alongside expedited approval mechanisms. This approach not only facilitates fast-track reimbursement and real-world data fit for use in these countries but also ensures that drug pricing reflects accurate revalidation and appropriate statistical methodologies.

### Limitations

This study has several limitations. First, while we aimed to accurately define patient groups using EMR data, these data can be unstructured and lack records of patients who were treated or died at other hospitals; however, we used EMR data converted to, and validated with, the OMOP CDM and matched both groups to ensure comparability using LASSO and negative control outcomes to reduce potential biases (eg, missingness from a selection bias perspective). Using this EMR data, the control group was defined as patients with DM who visited a tertiary care hospital, who may represent a more severe cohort than the general population of patients with DM. This may lead to an overestimation of economic burden in the control group. To address this limitation, we made efforts to reduce selection bias in this study, and our analysis demonstrated that the incremental economic burden of patients with DME was consistent and substantial across all cost categories and HRU over 3 years. Second, many patients had zero hospitalizations and zero hospital days. We used the zero-inflation model and selected variables by backward elimination because of the small variation in covariates across groups. When we add full variables in a negative binomial model without a zero-inflation model, the group value for the inpatient visits remained consistently significant, while the added unmatched covariates were not significant in any of the models. Third, we lacked data on the type and status of patient insurance (however, in South Korea, national health insurance services cover the majority of the population, and only 3% of patients are supported by medical aid programs). Nevertheless, our study provides unique real-world evidence of the economic burden from the perspective of patients with DME, showing that DME is associated with a significant economic burden compared with DM alone, highlighting the need for health care resource allocation and policy planning to address this issue. These findings may be of interest to health care providers, policy makers, and payers in their efforts to manage and reduce the economic impact of these conditions. Finally, in this study, we included the direct medical costs measurable from a single-center EMR. However, we did not include data from other centers, direct nonmedical costs (such as transportation costs), and indirect costs (such as productivity loss costs). Consequently, the patients’ economic burden might be underestimated.

### Further Research for Patients With DME

Further research is needed. First, a detailed analysis of the underlying economic burden of patients with DME should be conducted, which should include a breakdown of medical costs by source, such as medications, procedures, and tests. This comprehensive approach will provide valuable insights into specific areas that contribute to the economic burden beyond the scope of payer and entity analysis. In particular, leveraging the OMOP CDM with HERMES will facilitate the seamless generation of real-world evidence, supporting evidence-based decision-making in clinical, economic, and regulatory contexts with validated patient-centered data [33]. Second, further research could analyze the correlation between policy measures, such as the strengthening of coverage for severe diseases in South Korea, and their impact on economic and clinical outcomes related to nonreimbursement and hospitalization. Understanding these policy correlations will shed light on potential interventions and strategies to optimize patient care, reduce financial stress, and improve clinical outcomes. In addition, further research is needed to enhance our understanding of, and preparedness for managing, the care cycle of patients with DME. Specifically, investigations focusing on patient measurements, subsequent therapy, and factors contributing to the sudden worsening of symptoms can provide valuable insights into optimizing patient care and resource allocation. We observed that the speed of cost growth increased at 700 and 1000 days in the group with DME than that in the group with DM, which could be due to the aforementioned factors.

Finally, future research urgently needs to provide policy rationale and recommendations using appropriate statistical methods aimed at reducing the clinical economic burden on both patients and the healthcare system, while preventing pharmaceutical companies from skipping or delaying drug reimbursement in specific countries; for example, there is a need for future research to reach consensus on statistical methods for appropriately handling discrepancies between the results from randomized clinical trials and real-world evidence, as well as on how to incorporate these methods into economic evaluations for drug-cost regulation.

### Conclusions

Our study findings indicate that DME, even in the absence of PDR, is associated with significantly higher healthcare costs and HRU than DM alone. This underscores the substantial economic burden DME places on patients. Regulatory and economic policies should prioritize the implementation and reimbursement of innovative drugs and technology to address the challenges presented by DME, prevent vision deterioration, and mitigate the high economic burden on society. These insights have significant implications for healthcare providers and policy makers, highlighting the crucial need for the enhanced surveillance and management of patients with DR. By prioritizing disease progression prevention and mitigating associated healthcare costs, we can effectively improve patient outcomes and allocate healthcare resources more efficiently to reduce financial stress in patients with DME.

## Appendix

Multimedia Appendix 1 The 3-year observed economic burden of patients with diabetic macular edema by reimbursement categorization and payment entity.

Multimedia Appendix 2 The 3-year observed healthcare resource utilization of patients with diabetic macular edema.

Multimedia Appendix 3 The 3-year observed healthcare resource utilization of patients with diabetic macular edema in ophthalmology.

## Abbreviations

CCI: Charlson Comorbidity Index
CDM: Common Data Model
DM: diabetes mellitus
DME: diabetic macular edema
DR: diabetic retinopathy
ECM: exponential conditional model
EMR: electronic medical record
HRU: healthcare resource utilization
LASSO: least absolute shrinkage and selection operator
LOS: length of stay
NPDR: nonproliferative diabetic retinopathy
OHDSI: Observational Health Data Sciences and Informatics
OMOP: Observational Medical Outcomes Partnership
PDR: proliferative diabetic retinopathy
SNUBH: Seoul National University Bundang Hospital
STROBE: Strengthening the Reporting of Observational Studies in Epidemiology
VEGF: vascular endothelial growth factor

## Data Availability

The data sets generated and analyzed during this study are not publicly available because the Observational Medical Outcomes Partnership Common Data Model is a distributed research network, and access to the data is restricted to internal private networks.

## References

[1] Jampol LM, Glassman AR, Sun J (2020). Evaluation and care of patients with diabetic retinopathy. N Engl J Med.

[2] Leasher JL, Bourne RR, Flaxman SR, Jonas JB, Keeffe J, Naidoo N, Pesudovs K, Price H, White RA, Wong TY, Resnikoff S, Taylor HR, Vision Loss Expert Group of the Global Burden of Disease Study (2016). Global estimates on the number of people blind or visually impaired by diabetic retinopathy: a meta-analysis from 1990 to 2010. Diabetes Care.

[3] Stitt AW, Curtis TM, Chen M, Medina RJ, McKay GJ, Jenkins A, Gardiner TA, Lyons TJ, Hammes HP, Simó R, Lois N (2016). The progress in understanding and treatment of diabetic retinopathy. Prog Retin Eye Res.

[4] Bresnick GH (1986). Diabetic macular edema. A review. Ophthalmology.

[5] Romero-Aroca P (2011). Managing diabetic macular edema: the leading cause of diabetes blindness. World J Diabetes.

[6] Singh A, Stewart JM (2009). Pathophysiology of diabetic macular edema. Int Ophthalmol Clin.

[7] Virgili G, Parravano M, Evans JR, Gordon I, Lucenteforte E (2018). Anti-vascular endothelial growth factor for diabetic macular oedema: a network meta-analysis. Cochrane Database Syst Rev.

[8] Figueira J, Henriques J, Carneiro Â, Marques-Neves C, Flores R, Castro-Sousa JP, Meireles A, Gomes N, Nascimento J, Amaro M, Silva R (2021). Guidelines for the management of center-involving diabetic macular edema: treatment options and patient monitorization. Clin Ophthalmol.

[9] Schmidt-Erfurth U, Garcia-Arumi J, Bandello F, Berg K, Chakravarthy U, Gerendas BS, Jonas J, Larsen M, Tadayoni R, Loewenstein A (2017). Guidelines for the management of diabetic macular edema by the European society of retina specialists (EURETINA). Ophthalmologica.

[10] Ross EL, Hutton DW, Stein JD, Bressler NM, Jampol LM, Glassman AR, Diabetic Retinopathy Clinical Research Network (2016). Cost-effectiveness of aflibercept, bevacizumab, and ranibizumab for diabetic macular edema treatment: analysis from the diabetic retinopathy clinical research network comparative effectiveness trial. JAMA Ophthalmol.

[11] Economic burden of illness in Canada, 2010. The Public Health Agency of Canada.

[12] Li Y, Xu J, Gu Y, Sun X, Dong H, Chen C (2022). The disease and economic burdens of esophageal cancer in China from 2013 to 2030: dynamic cohort modeling study. JMIR Public Health Surveill.

[13] Chen S, Kuhn M, Prettner K, Yu F, Yang T, Bärnighausen T, Bloom DE, Wang C (2023). The global economic burden of chronic obstructive pulmonary disease for 204 countries and territories in 2020-50: a health-augmented macroeconomic modelling study. Lancet Glob Health.

[14] Parker ED, Lin J, Mahoney T, Ume N, Yang G, Gabbay RA, ElSayed NA, Bannuru RR (2024). Economic costs of diabetes in the U.S. in 2022. Diabetes Care.

[15] Gascon P, Borget I, Comet A, Carton L, Matonti F, Dupont-Benjamin L (2022). Costs comparison of treating diabetic macular edema with aflibercept, ranibizumab or dexamethasone at 1 year in France (INVICOST study). Eur J Ophthalmol.

[16] Overhage JM, Ryan PB, Reich CG, Hartzema AG, Stang PE (2012). Validation of a common data model for active safety surveillance research. J Am Med Inform Assoc.

[17] Huser V, DeFalco FJ, Schuemie M, Ryan PB, Shang N, Velez M, Park RW, Boyce RD, Duke J, Khare R, Utidjian L, Bailey C (2016). Multisite evaluation of a data quality tool for patient-level clinical data sets. EGEMS (Wash DC).

[18] Choi K, Park SJ, Han S, Kim S, Suh HS HERMES: a health resources econometric analysis tool. Observational Health Data Sciences and Informatics.

[19] Manning WG, Mullahy J (2001). Estimating log models: to transform or not to transform?. J Health Econ.

[20] Happich M, Reitberger U, Breitscheidel L, Ulbig M, Watkins J (2008). The economic burden of diabetic retinopathy in Germany in 2002. Graefes Arch Clin Exp Ophthalmol.

[21] Lee LJ, Yu AP, Cahill KE, Oglesby AK, Tang J, Qiu Y, Birnbaum HG (2008). Direct and indirect costs among employees with diabetic retinopathy in the United States. Curr Med Res Opin.

[22] Shea AM, Curtis LH, Hammill BG, Kowalski JW, Ravelo A, Lee PP, Sloan FA, Schulman KA (2008). Resource use and costs associated with diabetic macular edema in elderly persons. Arch Ophthalmol.

[23] Heintz E, Wiréhn AB, Peebo BB, Rosenqvist U, Levin LÅ (2010). Prevalence and healthcare costs of diabetic retinopathy: a population-based register study in Sweden. Diabetologia.

[24] Gonder JR, Walker VM, Barbeau M, Zaour N, Zachau BH, Hartje JR, Li R (2014). Costs and quality of life in diabetic macular edema: Canadian burden of diabetic macular edema observational study (C-REALITY). J Ophthalmol.

[25] Zhang X, Low S, Kumari N, Wang J, Ang K, Yeo D, Yip CC, Tavintharan S, Sum CF, Lim SC (2017). Direct medical cost associated with diabetic retinopathy severity in type 2 diabetes in Singapore. PLoS One.

[26] Jeon HL, Lee H, Yoon D, Lee Y, Kim JH, Jee D, Shin J (2020). Burden of diabetic macular oedema in patients receiving antivascular endothelial growth factor therapy in South Korea: a healthcare resource use and cost analysis. BMJ Open.

[27] Moshfeghi AA, Lanitis T, Kropat G, Kuznik A, Gibson A, Feng H, Prenner J (2020). Social cost of blindness due to AMD and diabetic retinopathy in the United States in 2020. Ophthalmic Surg Lasers Imaging Retina.

[28] Chen EM, Chen D, Chilakamarri P, Lopez R, Parikh R (2021). Economic challenges of artificial intelligence adoption for diabetic retinopathy. Ophthalmology.

[29] Chen E, Looman M, Laouri M, Gallagher M, Van Nuys K, Lakdawalla D, Fortuny J (2010). Burden of illness of diabetic macular edema: literature review. Curr Med Res Opin.

[30] Mulligan K, Kim J, Tysinger B, Blim J, Emerson G, Ferrone P, Kim JE, Seabury S, Hahn P (2023). The broader economic value of treatment for diabetic macular edema. Diabetes Care.

[31] Jaki Mekjavić P, Jūratė Balčiūnienė V, Ćeklić L, Ernest J, Jamrichova Z, Zsolt Nagy Z, Petkova I, Teper S, Gardašević Topčić I, Veith M (2019). The burden of macular diseases in central and eastern Europe-implications for healthcare systems. Value Health Reg Issues.

[32] Lai TT, Hsieh YT, Yang CM, Ho TC, Yang CH (2020). Effect of reimbursement policy on visual outcomes in patients with diabetic macular edema treated with ranibizumab. Retina.

[33] Choi K, Park SJ, Han S, Mun Y, Lee DY, Chang D, Kim S, Yoo S, Woo SJ, Park KH, Suh HS (2023). Patient-centered economic burden of exudative age-related macular degeneration: retrospective cohort study. JMIR Public Health Surveill.

