# Supplementary material for: Patient-Centered Economic Burden of Diabetic Macular Edema: Retrospective Cohort Study
Source: JMIR Public Health Surveill. 2024 Oct 8;10:e56741. doi: 10.2196/56741 (PMC11496919; doi:10.2196/56741)
Supplement: Multimedia Appendix 1 [file publichealth_v10i1e56741_app1.docx]

|  |  | Group | N | Mean | Standard deviation | Median | Skewness | Kurtosis |
| --- | --- | --- | --- | --- | --- | --- | --- | --- |
| Three-year economic burden | Total cost | DM | 1460 | 2103.69 | 3645.19 | 1163.12 | 6.42 | 62.18 |
|  |  | DME | 450 | 4478.39 | 5105.24 | 2698.12 | 2.81 | 13.25 |
|  | Reimbursement cost | DM | 1460 | 1477.38 | 2845.15 | 798.47 | 7.57 | 85.1 |
|  |  | DME | 450 | 2878.94 | 4132.03 | 1473.21 | 3.63 | 21.33 |
|  | Non-reimburse-ment cost | DM | 1460 | 626.31 | 1230.62 | 237.18 | 7.01 | 78.22 |
|  |  | DME | 450 | 1599.46 | 1664.98 | 1044.88 | 2.04 | 5.27 |
|  | Cost covered by insurance | DM | 1460 | 900.77 | 2328.1 | 323.25 | 9.25 | 123.45 |
|  |  | DME | 450 | 1902.25 | 3282.7 | 640 | 3.42 | 16.6 |
|  | Out-of-pocket  cost | DM | 1460 | 1202.92 | 1703.77 | 728.8 | 5.11 | 42.42 |
|  |  | DME | 450 | 2576.15 | 2453.53 | 1791.57 | 1.9 | 4.87 |
| First year | Total cost | DM | 1460 | 981.04 | 1233.78 | 495.53 | 2.31 | 5.52 |
|  |  | DME | 450 | 2135.13 | 2097.05 | 1435.9 | 1.97 | 4.83 |
|  | Reimbursement cost | DM | 1460 | 675.81 | 869.71 | 368.75 | 2.61 | 7.68 |
|  |  | DME | 450 | 1296.95 | 1690.87 | 620.21 | 2.12 | 4.45 |
|  | Non-reimburse-ment cost | DM | 1460 | 305.23 | 508.92 | 108.33 | 3.3 | 15.45 |
|  |  | DME | 450 | 838.18 | 740.56 | 627.4 | 1.58 | 3.08 |
|  | Cost covered by insurance | DM | 1460 | 398.41 | 652.61 | 148.76 | 2.98 | 9.9 |
|  |  | DME | 450 | 853.12 | 1403.74 | 223.55 | 2.36 | 5.19 |
|  | Out-of-pocket  cost | DM | 1460 | 582.64 | 685.81 | 328.61 | 2.36 | 6.74 |
|  |  | DME | 450 | 1282.01 | 1068.63 | 1020.86 | 2.25 | 9.46 |
| Second year | Total cost | DM | 1460 | 562.34 | 1950.47 | 150.82 | 10.66 | 147.86 |
|  |  | DME | 450 | 1293.64 | 2374.96 | 448.66 | 3.98 | 21.36 |
|  | Reimbursement cost | DM | 1460 | 404 | 1593.11 | 101.7 | 11.43 | 161.59 |
|  |  | DME | 450 | 867.21 | 1923.62 | 246.55 | 5.09 | 34.91 |
|  | Non-reimburse-ment cost | DM | 1460 | 158.34 | 523.48 | 0 | 7.41 | 73.04 |
|  |  | DME | 450 | 426.43 | 746.97 | 119.04 | 3.29 | 14.94 |
|  | Cost covered by insurance | DM | 1460 | 255.12 | 1363.69 | 36.57 | 12.74 | 197.27 |
|  |  | DME | 450 | 583.28 | 1502.6 | 88.88 | 4.91 | 31.25 |
|  | Out-of-pocket  cost | DM | 1460 | 307.22 | 746.79 | 91.88 | 7.03 | 73.5 |
|  |  | DME | 450 | 710.36 | 1088.12 | 295.64 | 2.78 | 9.74 |
| Third year | Total cost | DM | 1460 | 560.3 | 2004.92 | 92.23 | 8.5 | 92.22 |
|  |  | DME | 450 | 1049.63 | 2049.9 | 260.98 | 4.54 | 31.08 |
|  | Reimbursement cost | DM | 1460 | 397.56 | 1471.88 | 54.54 | 8.24 | 79.51 |
|  |  | DME | 450 | 714.78 | 1655.7 | 164.61 | 5.54 | 43.57 |
|  | Non-reimburse-ment cost | DM | 1460 | 162.75 | 811.77 | 0 | 15.25 | 291.14 |
|  |  | DME | 450 | 334.85 | 641.89 | 23.26 | 3.28 | 15.05 |
|  | Cost covered by insurance | DM | 1460 | 247.24 | 1179.03 | 16.2 | 9.47 | 106.53 |
|  |  | DME | 450 | 465.85 | 1269.8 | 52.28 | 5.78 | 45.89 |
|  | Out-of-pocket  cost | DM | 1460 | 313.06 | 1049.12 | 54.67 | 11.81 | 196.72 |
|  |  | DME | 450 | 583.78 | 962.17 | 182.54 | 2.97 | 12.39 |
