# Supplementary material for: Patient-Centered Economic Burden of Diabetic Macular Edema: Retrospective Cohort Study
Source: JMIR Public Health Surveill. 2024 Oct 8;10:e56741. doi: 10.2196/56741 (PMC11496919; doi:10.2196/56741)
Supplement: Multimedia Appendix 2 [file publichealth_v10i1e56741_app2.docx]

|  |  | Group | N | Mean | Standard deviation | Median |
| --- | --- | --- | --- | --- | --- | --- |
| Three-year health resource utilization | Outpatient visit | DM | 1460 | 9.71 | 11.49 | 6 |
|  |  | DME | 450 | 18.35 | 17.07 | 13.5 |
|  | Inpatient visit | DM | 1460 | 0.25 | 0.63 | 0 |
|  |  | DME | 450 | 0.44 | 0.95 | 0 |
|  | Emergency department visit | DM | 1460 | 0 | 0.06 | 0 |
|  |  | DME | 450 | 0 | 0 | 0 |
|  | Laboratory visit | DM | 1460 | 3.83 | 7.82 | 0 |
|  |  | DME | 450 | 1.76 | 4.12 | 0 |
|  | Length of stay | DM | 1460 | 1.4 | 5.55 | 0 |
|  |  | DME | 450 | 1.98 | 8.6 | 0 |
| First year | Outpatient visit | DM | 1460 | 4.88 | 5.78 | 3 |
|  |  | DME | 450 | 9.08 | 8.47 | 8 |
|  | Inpatient visit | DM | 1460 | 0.17 | 0.47 | 0 |
|  |  | DME | 450 | 0.22 | 0.59 | 0 |
|  | Emergency department visit | DM | 1460 | 0 | 0.05 | 0 |
|  |  | DME | 450 | 0 | 0 | 0 |
|  | Laboratory visit | DM | 1460 | 1.38 | 3.99 | 0 |
|  |  | DME | 450 | 0.61 | 2.46 | 0 |
|  | Length of stay | DM | 1460 | 0.68 | 2.51 | 0 |
|  |  | DME | 450 | 0.7 | 2.33 | 0 |
| Second year | Outpatient visit | DM | 1460 | 2.65 | 5.03 | 0 |
|  |  | DME | 450 | 4.95 | 6.59 | 2 |
|  | Inpatient visit | DM | 1460 | 0.04 | 0.27 | 0 |
|  |  | DME | 450 | 0.12 | 0.44 | 0 |
|  | Emergency department visit | DM | 1460 | 0 | 0.03 | 0 |
|  |  | DME | 450 | 0 | 0 | 0 |
|  | Laboratory visit | DM | 1460 | 1.32 | 4.58 | 0 |
|  |  | DME | 450 | 0.72 | 2.42 | 0 |
|  | Length of stay | DM | 1460 | 0.33 | 2.83 | 0 |
|  |  | DME | 450 | 0.76 | 5.03 | 0 |
| Third year | Outpatient visit | DM | 1460 | 2.17 | 4.54 | 0 |
|  |  | DME | 450 | 4.32 | 6.79 | 0.5 |
|  | Inpatient visit | DM | 1460 | 0.04 | 0.24 | 0 |
|  |  | DME | 450 | 0.1 | 0.44 | 0 |
|  | Emergency department visit | DM | 1460 | 0 | 0.04 | 0 |
|  |  | DME | 450 | 0 | 0 | 0 |
|  | Laboratory visit | DM | 1460 | 1.13 | 3.65 | 0 |
|  |  | DME | 450 | 0.44 | 1.8 | 0 |
|  | Length of stay | DM | 1460 | 0.39 | 3.43 | 0 |
|  |  | DME | 450 | 0.52 | 3.56 | 0 |
