# Supplementary material for: Patient-Centered Economic Burden of Diabetic Macular Edema: Retrospective Cohort Study
Source: JMIR Public Health Surveill. 2024 Oct 8;10:e56741. doi: 10.2196/56741 (PMC11496919; doi:10.2196/56741)
Supplement: Multimedia Appendix 3 [file publichealth_v10i1e56741_app3.docx]

|  |  | Group | N | Mean | Standard deviation | Median |
| --- | --- | --- | --- | --- | --- | --- |
| Three-year health resource utilization | Outpatient visit | DM | 1460 | 1.48 | 3.07 | 0 |
|  |  | DME | 450 | 11.36 | 10.76 | 8 |
|  | Inpatient visit | DM | 1460 | 0.08 | 0.34 | 0 |
|  |  | DME | 450 | 0.27 | 0.64 | 0 |
|  | Emergency department visit | DM | 1460 | 0 | 0.06 | 0 |
|  |  | DME | 450 | 0 | 0 | 0 |
|  | Laboratory visit | DM | 1460 | 0.16 | 0.71 | 0 |
|  |  | DME | 450 | 0.92 | 2.23 | 0 |
|  | Length of stay | DM | 1460 | 0.1 | 0.49 | 0 |
|  |  | DME | 450 | 0.5 | 1.4 | 0 |
| First year | Outpatient visit | DM | 1460 | 0.88 | 1.93 | 0 |
|  |  | DME | 450 | 6.34 | 5.87 | 6 |
|  | Inpatient visit | DM | 1460 | 0.07 | 0.33 | 0 |
|  |  | DME | 450 | 0.17 | 0.51 | 0 |
|  | Emergency department visit | DM | 1460 | 0 | 0.05 | 0 |
|  |  | DME | 450 | 0 | 0 | 0 |
|  | Laboratory visit | DM | 1460 | 0.09 | 0.55 | 0 |
|  |  | DME | 450 | 0.39 | 1.49 | 0 |
|  | Length of stay | DM | 1460 | 0.09 | 0.47 | 0 |
|  |  | DME | 450 | 0.36 | 1.29 | 0 |
| Second year | Outpatient visit | DM | 1460 | 0.34 | 1.07 | 0 |
|  |  | DME | 450 | 2.86 | 4.07 | 1 |
|  | Inpatient visit | DM | 1460 | 0 | 0.03 | 0 |
|  |  | DME | 450 | 0.06 | 0.27 | 0 |
|  | Emergency department visit | DM | 1460 | 0 | 0.03 | 0 |
|  |  | DME | 450 | 0 | 0 | 0 |
|  | Laboratory visit | DM | 1460 | 0.04 | 0.29 | 0 |
|  |  | DME | 450 | 0.29 | 1.23 | 0 |
|  | Length of stay | DM | 1460 | 0 | 0.05 | 0 |
|  |  | DME | 450 | 0.07 | 0.39 | 0 |
| Third year | Outpatient visit | DM | 1460 | 0.25 | 0.94 | 0 |
|  |  | DME | 450 | 2.16 | 3.62 | 0 |
|  | Inpatient visit | DM | 1460 | 0 | 0.08 | 0 |
|  |  | DME | 450 | 0.05 | 0.28 | 0 |
|  | Emergency department visit | DM | 1460 | 0 | 0.04 | 0 |
|  |  | DME | 450 | 0 | 0 | 0 |
|  | Laboratory visit | DM | 1460 | 0.03 | 0.25 | 0 |
|  |  | DME | 450 | 0.23 | 1.07 | 0 |
|  | Length of stay | DM | 1460 | 0.01 | 0.12 | 0 |
|  |  | DME | 450 | 0.06 | 0.39 | 0 |
